# Distinct Cell Surface Expression Patterns of N-Glycosylation Site Mutants of AMPA-Type Glutamate Receptor under the Homo-Oligomeric Expression Conditions

**DOI:** 10.3390/ijms21145101

**Published:** 2020-07-19

**Authors:** Jyoji Morise, Saki Yamamoto, Ryosuke Midorikawa, Kogo Takamiya, Motohiro Nonaka, Hiromu Takematsu, Shogo Oka

**Affiliations:** 1 Department of Biological Chemistry, Human Health Sciences, Graduate School of Medicine, Kyoto University, Kyoto 606-8501, Japan; morise.jyoji.2n@kyoto-u.ac.jp (J.M.); yymrchimnss@gmail.com (S.Y.); nonaka.motohiro.4r@kyoto-u.ac.jp (M.N.); htakema@fujita-hu.ac.jp (H.T.); 2 Department of Integrative Physiology, Faculty of Medicine, University of Miyazaki, Miyazaki 889-1692, Japan; ryosuke_midorikawa@med.miyazaki-u.ac.jp (R.M.); takamiya@med.miyazaki-u.ac.jp (K.T.); 3 Department of Molecular Cell Biology, Faculty of Medical Technology, Graduate School of Health Sciences, Fujita Health University, Aichi 470-1192, Japan

**Keywords:** AMPA-type glutamate receptor, GluA1, GluA2, N-glycan

## Abstract

The AMPA-type glutamate receptor (AMPAR) is a homotetrameric or heterotetrameric ion channel composed of various combinations of four subunits (GluA1–4), and its abundance in the synapse determines the strength of synaptic activity. The formation of oligomers in the endoplasmatic reticulum (ER) is crucial for AMPAR subunits’ ER-exit and translocation to the cell membrane. Although N-glycosylation on different AMPAR subunits has been shown to regulate the ER-exit of hetero-oligomers, its role in the ER-exit of homo-oligomers remains unclear. In this study, we investigated the role of N-glycans at GluA1N63/N363 and GluA2N370 in ER-exit under the homo-oligomeric expression conditions, whose mutants are known to show low cell surface expressions. In contrast to the N-glycosylation site mutant GluA1N63Q, the cell surface expression levels of GluA1N363Q and GluA2N370Q increased in a time-dependent manner. Unlike wild-type (WT) GluA1, GluA2WT rescued surface GluA2N370Q expression. Additionally, the expression of GluA1N63Q reduced the cell surface expression level of GluA1WT. In conclusion, our findings suggest that these N-glycans have distinct roles in the ER-exit of GluA1 and GluA2 homo-oligomers; N-glycan at GluA1N63 is a prerequisite for GluA1 ER-exit, whereas N-glycans at GluA1N363 and GluA2N370 control the ER-exit rate.

## 1. Introduction

The AMPA-type glutamate receptor (AMPAR) is an ionotropic glutamate receptor involved in fast excitatory synaptic transmission in the central nervous system [1]. Its abundance on the neuronal cell surface determines synaptic plasticity and contributes to memory formation and learning. AMPAR functions as a heterotetramer or homotetramer composed of various combinations of four subunits, GluA1–4 [2]. The majority of AMPAR subunits are stored in the endoplasmatic reticulum (ER) [3], and the regulation of AMPAR abundance on the neuronal cell surface is crucial for synaptic plasticity. Since GluA2 determines a calcium impermeability of AMPAR [4], the GluA2-containing heterotetramer is calcium-impermeable. In contrast, the GluA1 homotetramer is calcium-permeable. Most of the AMPARs in neurons function in the form of GluA2-containing heterotetramers [5]. However, the importance of calcium-permeable AMPARs in synaptic plasticity has also been recently reported [6,7,8]. Therefore, it is important to elucidate the mechanism of ER-exit in the homo-oligomeric combination in order to comprehensively understand the role of the homotetramer. AMPAR subunits’ oligomerization defines its ability to translocate from the endoplasmatic reticulum (ER) to the cell surface [9,10]. Importantly, in the absence of the N-terminal domain (NTD), which is essential for oligomerization [11], AMPAR abundance on the cell surface is significantly reduced [12].

GluA1 and GluA2 are the primary AMPAR subunits in neurons [5]. The extracellular domains of these subunits contain several N-glycosylation sites (N63, N249, N257, N363, N401, and N406 for GluA1 and N256, N370, N406, and N413 for GluA2; Figure S1) [13]. GluA1 and GluA2 transported to the cell surface membrane have complex-type N-glycans [14,15]. Therefore, AMPAR subunits are likely to pass through a typical Golgi apparatus-dependent pathway. We previously showed that GluA2 contains the human natural killer-1 (HNK-1) epitope at N413 [16,17], and the HNK-1 epitope on GluA2 is crucial for AMPAR cell surface expression and spine maturation [18]. Notably, mice lacking the HNK-1 epitope exhibited learning and memory formation impairments, as well as reduced long-term potentiation in the hippocampal CA1 region [19], indicating that N-glycosylation of AMPAR is indispensable for synaptic plasticity. By establishing N-glycosylation site mutants, we found that N-glycans at N63 and N363 of GluA1 were essential for GluA1 homotetramerization and cell surface expression [20]. Similarly, the inhibition of N-glycosylation at N370 of GluA2, a site homologous to N363 of GluA1 [13], reduced the cell surface expression (Figure S1) [17], highlighting the importance of N-glycan at N363 for AMPAR subunit transport from the ER to the cell surface. By contrast, under the hetero-oligomeric expression conditions of GluA1 and GluA2, in which one subunit was mutated and the other was wild-type (WT), the GluA2N370 mutation significantly reduced the cell surface expression of GluA1WT [17]; GluA2WT rescued the effects of GluA1N363 mutation [20]. These results suggest a potential role for N-glycans in the regulation of the ER-exit of hetero-oligomers.

Since GluA1 and GluA2 also form homo-oligomers [2,21,22], in this study, we examined the role of N-glycans in GluA1 and GluA2 under the homo-oligomeric expression conditions. We generated different tagged WT and N-glycosylation site mutants and assessed the cell surface expression of GluA1 and GluA2 using a combination of WT and its mutant by immunostaining. We found that N-glycan at N63 of GluA1 is essential for the ER-exit of homo-oligomers and that N-glycans at N363 of GluA1 and N370 of GluA2 affect the ER-exit rate of homo-oligomers, suggesting that specific N-glycans have a distinct role in the ER-exit of GluA1 and GluA2 homo-oligomers.

## 2. Results

### 2.1. Distinct Roles of GluA1 and GluA2 N-Glycans on Cell Surface Expression Levels

To determine the role of certain N-glycans in the ER-exit of GluA1 and GluA2 homo-oligomers, we assessed the cell surface expression levels of GluA1 and GluA2 mutants when co-expressed with WT subunits. Note that the occupancy of GluA1 N-glycosylation sites was approximately 100%, except for the N401 site [23]. Likewise, the occupancy of all GluA2 N-glycosylation sites is likely to be approximately 100% because respective mutants showed lower molecular wight than WT [17]. Since all asparagine in the consensus sequence (N-X-S/T) of GluA1 and GluA2, except for GluA1N401, which is not used in this study, is N-glycosylated, the N-glycosylation site mutants can clarify the role of respective N-glycans in ER-exit. To this end, we labeled GluA1 and GluA2 subunits by fusing them with green fluorescent protein (GFP) at the C-terminus (GluA1-GFP and GluA2-GFP) or by adding HA-tag and myc-tag at the N-terminus and C-terminus, respectively (HA-GluA1-myc and HA-GluA2-myc). It has been previously shown that these tags at these specific positions are unlikely to affect AMPAR formation or trafficking [24,25,26,27].

**Figure 1:**
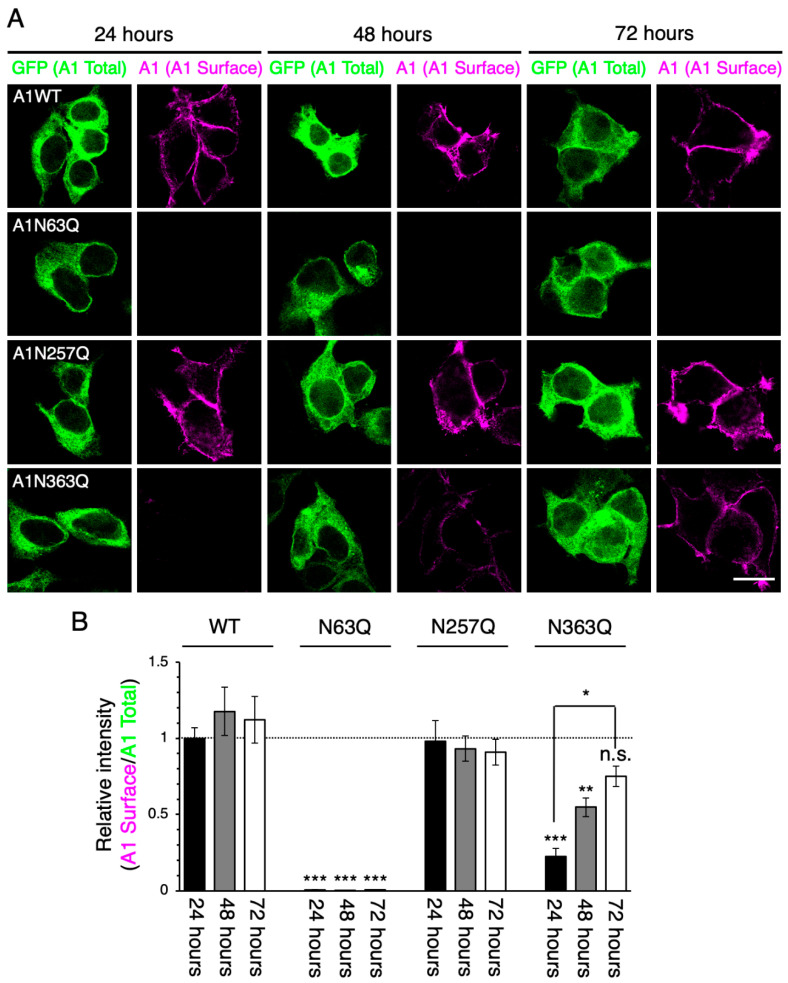
Cell surface expression levels of GluA1N363Q increase in a time-dependent manner. (**A**) Representative immunostaining images showing the cell surface expression levels of wild-type (WT) and mutant (N63Q, N257Q, and N363Q) GluA1. HEK293 cells were stained using an anti-GluA1 mAb (magenta; pseudocolor). The fluorescence intensity of green fluorescent protein (GFP; shown in green) indicates the total expression levels of individual GluA1 subunits. Scale bar, 20 µm. (**B**) Relative intensities of cell surface GluA1 WT and mutants at 24, 48, and 72 h post-transfection. The fluorescence intensity of surface GluA1 was normalized to that of GFP and GluA1WT at 24 h post-transfection. ***, *p* < 0.001; **, *p* < 0.01; *, *p* < 0.05; n.s., *p* > 0.05. Error bars represent the standard error of the mean (SEM).

**Figure 2:**
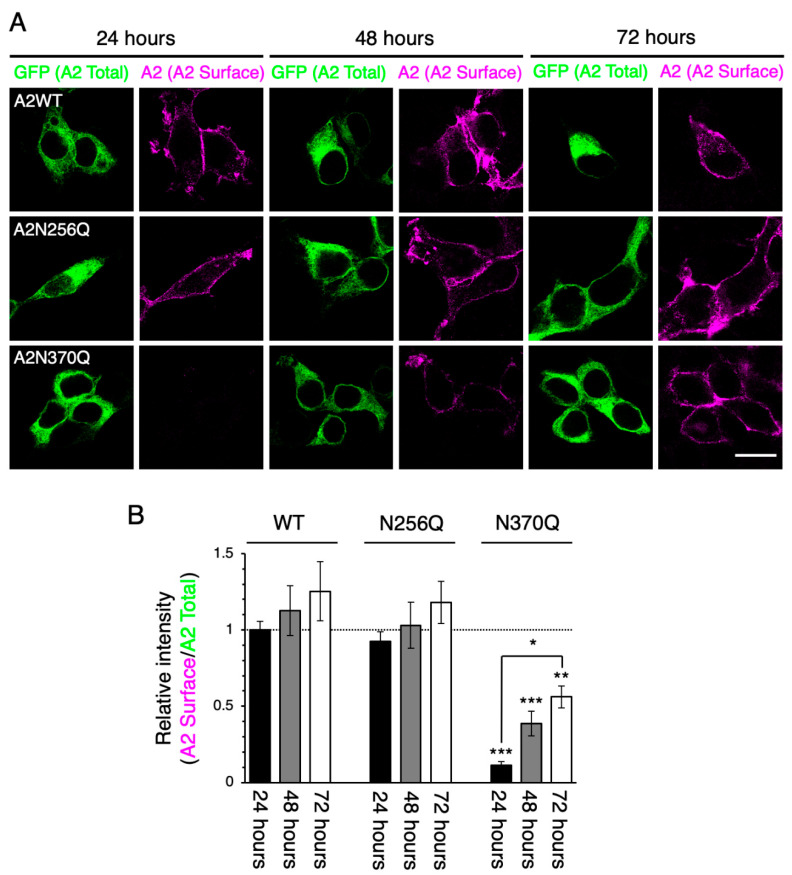
Cell surface expression levels of GluA2N370Q increase in a time-dependent manner. (**A**) Representative immunostaining images showing the cell surface expression levels of WT and mutant (N256Q and N370Q) GluA2. HEK293 cells were stained using an anti-GluA2 mAb (magenta; pseudocolor). The fluorescence intensity of GFP (shown in green) indicates the total expression levels of individual GluA2 subunits. Scale bar, 20 µm. (**B**) Relative intensities of cell surface WT and mutant GluA2 at 24, 48, and 72 h post-transfection. The fluorescent intensity of surface GluA2 was normalized to that of GFP and GluA2WT at 24 h post-transfection. ***, *p* < 0.001; **, *p* < 0.01; *, *p* < 0.05. Error bars represent the SEM.

Among the GluA1 and GluA2 N-glycosylation site mutants, the cell surface expression levels of N63 and N363 mutants for GluA1 and the N370 mutant for GluA2 were considerably lower than WT or other N-glycosylation mutants in HEK293 cells at 24–48 h post-transfection [17,20]. Consistent with these previous reports, we detected GluA1WT and GluA1N257Q on the cell surface at 24 h post-transfection (Figure 1). Similar results were obtained for GluA2WT and GluA2N256Q (Figure 2), suggesting that the C-terminal GFP did not affect the cell surface expression of GluA1 or GluA2. Additionally, the cell surface expression levels of GluA1N63Q, GluA1N363Q, and GluA2N370Q were significantly suppressed at 24 h and 48 h post-transfection compared with those of the WT subunits (Figure 1), as previously reported [17,20].

To assess the long-term effects of N-glycan defects in the ER-exit of GluA1 and GluA2 homo-oligomers, we assessed the cell surface expression levels of GluA1 and GluA2 mutants at 72 h post-transfection. Although we could not detect GluA1N63Q on the cell surface at 72 h post-transfection (Figure 1), the levels of GluA1N363Q on the cell surface were similar to those of GluA1WT. While cell surface expression levels of GluA2N370Q were lower than those of GluA2WT at 72 h post-transfection, the levels of GluA2N370Q on the cell surface at 72 h post-transfection were significantly higher than those at 24 h post-transfection (Figure 2). These results suggested that the cell surface expression levels of GluA1N363Q and GluA2N370Q, but not GluA1N63Q, increased in a time-dependent manner. Since GluA1N363 is highly homologous to GluA2N370 (Figure S1), we believe that GluA1 and GluA2 N-glycans at these positions regulate the anterograde ER transport rate of homo-oligomers.

### 2.2. N-Glycan at GluA1N63 Is Essential for GluA1 Cell Surface Expression

It has been reported that GluA2WT co-expression rescues the cell surface expression of GluA1N363Q at 24 h post-transfection [20]. By contrast, GluA2N370Q reduced GluA1WT cell surface expression levels [17]. Hence, GluA2 N-glycans are believed to control the ER-exit rate of GluA1. Although the importance of specific N-glycans on the regulation of the ER-exit of hetero-oligomers is understood, it remains unclear which N-glycans are important for the ER-exit of homo-oligomers. To determine the role of certain N-glycans on the ER-exit of homo-oligomers, we assessed the cell surface expression levels of mutant subunits when co-expressed with WT subunits. We distinguished between WT GluA1 and its mutants by fusing them with different tags.

**Figure 3:**
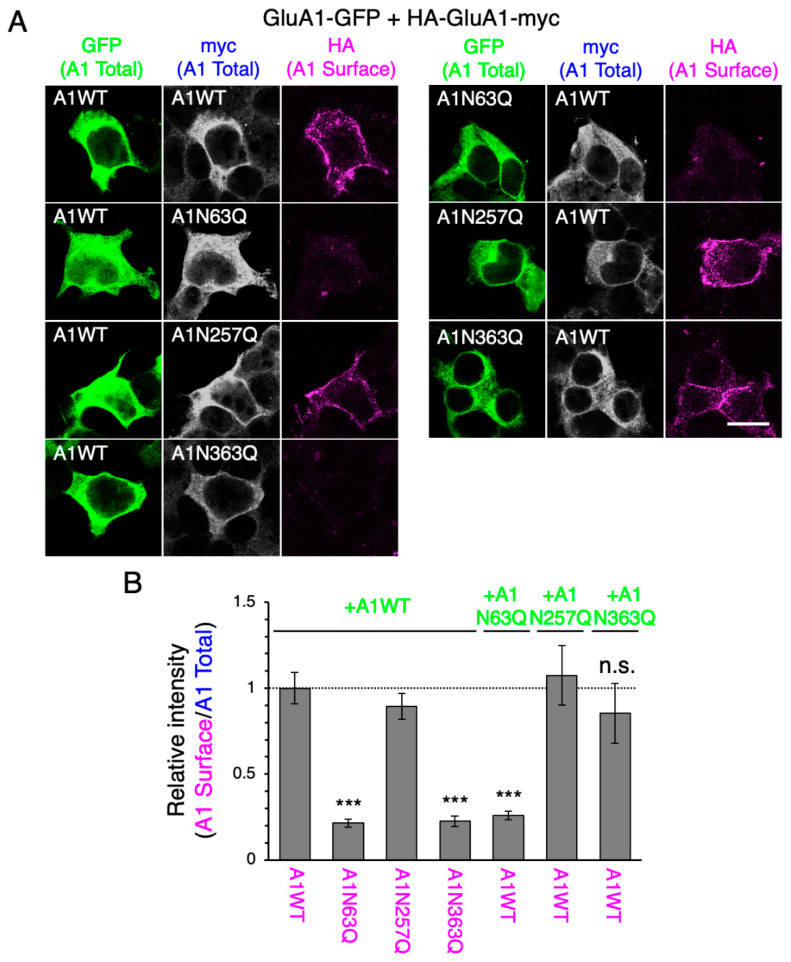
GluA1N63Q reduces the cell surface expression levels of GluA1WT. (**A**) Representative immunostaining images showing the cell surface levels of WT and mutant (N63Q, N257Q, and N363Q) GluA1 tagged with HA and myc (at the N- and C-terminus, respectively) in HEK293 cells co-expressing GFP-tagged WT or mutant GluA1 (green). Images were taken at 24 h post-transfection. Surface GluA1 was labeled using an anti-HA mAb (magenta; pseudocolor), and total GluA1 was detected using an anti-myc pAb (white; pseudocolor). Scale bar, 20 µm. (**B**) Relative cell surface levels of HA-GluA1-myc WT and mutants in cells co-expressing WT and mutant GluA1-GFP. The fluorescence intensity of HA was normalized to that of myc and homo-oligomeric combination of WTs at 24 h post-transfection. Low GluA1N63 cell surface expression levels were observed, possibly due to anti-HA mAb internalization before cell fixation. ***, *p* < 0.001; n.s., *p* > 0.05. Error bars represent the SEM.

After 24 h of co-transfection with GluA1WT-GFP, the cell surface expression levels of HA-GluA1N63Q-myc and HA-GluA1N363Q-myc were lower than those of the WT or the reference mutant N257Q (Figure 3). When GluA2WT-GFP was co-expressed, the surface levels of HA-GluA1N363Q-myc were increased, whereas those of HA-GluA1N63Q-myc remained low (Figure S2A,B). These findings were consistent with our previous report [20], confirming that the fusions of HA and myc tags did not affect AMPAR subunit trafficking. These results suggested that, unlike GluA2WT, GluA1WT did not enhance the ER-exit of N63Q or N363Q subunits. The cell surface expression levels of HA-GluA1WT-myc were decreased by GluA1N63Q-GFP, but not GluA1N363Q-GFP, co-expression (Figure 3). On the other hand, these mutants did not affect the cell surface expression of GluA2WT (Figure S2C,D), consistent with previous findings [20]. Taken together, the presence of GluA1WT did not affect the ER-exit of the N63Q and N363Q mutants; GluA1WT was transported to the cell surface even in the presence of N363Q. However, N63Q suppressed GluA1WT ER-exit. Hence, the presence of GluA1 subunits lacking N-glycan at N63 in the cell suppresses GluA1 transportation to the cell membrane regardless of the presence of fully glycosylated GluA1.

### 2.3. GluA2WT Enhances the ER-Exit Rate of GluA2N370Q

**Figure 4:**
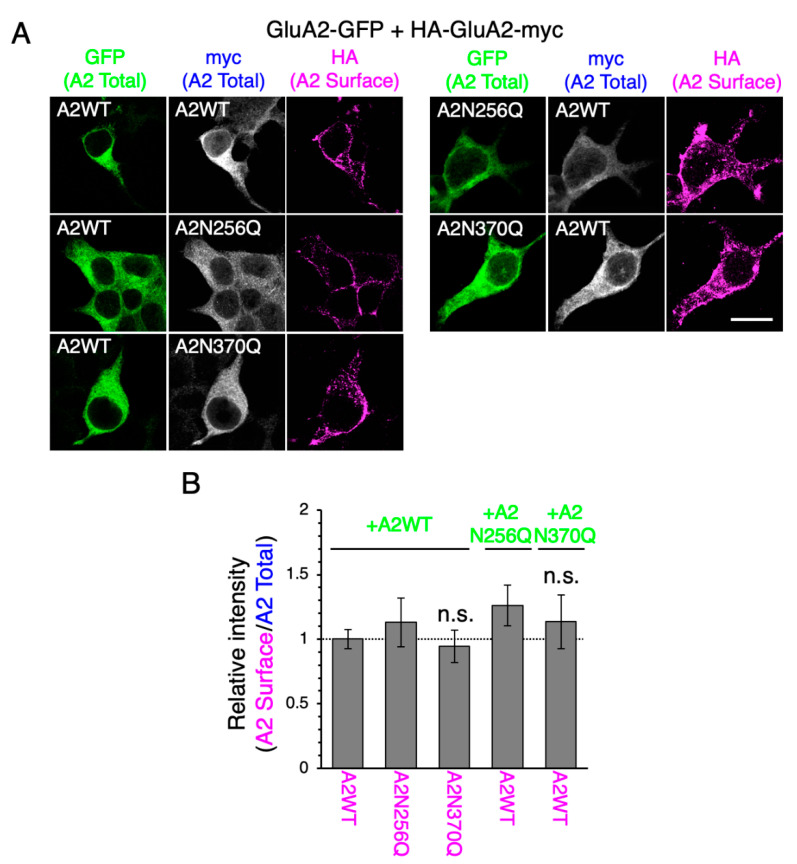
GluA2WT enhances the cell surface expression of GluA2N370Q. (**A**) Representative immunostaining images showing the cell surface WT and mutant (N256Q and N370Q) GluA2 tagged with HA and myc (at the N- and C-terminus, respectively) in HEK293 cells co-expressing GFP-tagged WT or mutant GluA2 (green). Images were taken at 24 h post-transfection. Surface GluA2 was labeled using an anti-HA mAb (magenta; pseudocolor) and total GluA2 was detected using an anti-myc pAb (white; pseudocolor). Scale bar, 20 µm. (**B**) Relative cell surface levels of WT and mutant HA-GluA2-myc in cells co-expressing WT and mutant GluA2-GFP. The fluorescence intensity of HA was normalized to that of myc and homo-oligomeric combination of WTs at 24 h post-transfection. n.s., *p* > 0.05. Error bars represent the SEM.

To investigate the relevance of GluA2 N-glycosylation in the ER-exit of GluA2 homo-oligomers, we expressed GluA2-GFP and HA-GluA2-myc into HEK293 cells. When GluA2WT-GFP was co-expressed, the levels of HA-GluA2N370Q-myc on the cell surface were similar to those of the WT or reference mutant N256Q (Figure 4). The cell surface expression levels of HA-GluA2WT-myc were not altered by GluA2N370Q-GFP co-expression. Although the expression of GluA2N370Q suppressed the transportation of GluA1WT to the cell surface (Figure S2A,B), GluA1WT did not affect GluA2N370Q cell surface expression levels (Figure S2C,D), as reported previously [17]. Collectively, these findings suggest that the presence of specific N-glycans on GluA2 initiatively regulates the ER-exit of homo-oligomers as well as hetero-oligomers.

## 3. Discussion

The importance of N-glycosylation in the regulation of cell surface expression of GluA1 and GluA2 under the hetero-oligomeric expression conditions has been previously demonstrated [17,20]. In this study, we assessed the role of specific N-glycans on the cell surface levels under the homo-oligomer forming conditions using different combinations of WT and mutants. We found that the cell surface levels of GluA1N363Q and GluA2N370Q, but not GluA1N63Q, increased in a time-dependent manner (Figure 1 and Figure 2). One possibility for the slow appearance of GluA1N363Q and GluA2N370Q is that the cell surface expression is apparently low due to the rapid translocation from the cell surface to an intracellular compartment. Meanwhile, we previously investigated the agonist-induced internalization level of AMPAR subunits by using a biotinylation assay [20]. Unlike the GluA1WT, we could not detect the internalized GluA1N363 mutant, indicating that the GluA1N363 mutant is not rapidly incorporated into the intracellular matrix. Taken together, we concluded that GluA1N363 and GluA2N370 mutants shows delayed ER-exit. Moreover, we previously provided evidence that GluA1N63Q and GluA1N363Q were degraded in lysosomes [20]. However, since GluA1N363Q exhibited long-term cell surface expression (Figure 1), we believe that only a portion of the mutant subunits is degraded in the lysosomes.

By contrast, we could not detect GluA1N63Q on the cell surface, even after long-term cell culture (Figure 1). Additionally, the GluA1N63Q cell surface expression level did not increase, even after co-expressing GluA1WT or GluA2WT (Figure 2; Figure S2). These findings suggest that GluA1N63Q subunits are more prone to lysosomal degradation than GluA1N363Q subunits. Since both GluA1N63Q and GluA1N363Q retained their ability to interact with GluA2WT [17], it is unlikely that these mutants have conformational aberrations. Instead, GluA1N63Q could be transported to the lysosomes by lysosomal pathway components. Interestingly, the expression of GluA1N63Q inhibited GluA1WT transportation to the cell surface (Figure 2). Future studies are required to assess whether GluA1WT is also transported to the lysosomes for degradation, as well as which lysosomal pathway components are involved in this process.

Although both GluA1N363Q and GluA2N370Q exhibited long-term cell surface expression, their behavior in the presence of GluA1 or GluA2 WT subunit differed. This finding could be explained by the fact that GluA2 is the dominant subunit regulating the anterograde transport of AMPAR [17], which is further supported by the fact that GluA2 but not GluA1 rescued the surface expression of GluA1N363Q and GluA2N370Q (Figure 4; Figure S2C,D). Interestingly, GluA2N370Q expression decreased the cell surface expression levels of GluA1WT. By contrast, the expression of GluA1N363Q did not affect the cell surface expression levels of GluA1WT. The ability of AMPAR subunits to form oligomers in the ER is believed to be important for ER-exit [9,10]. Based on the findings of this study, we believe that the ability of GluA1 to recognize other AMPAR subunits to form oligomers in the ER might be weaker than that of GluA2. However, since GluA1N63Q suppressed GluA1WT ER-exit, it is likely that the absence of N-glycan at N63 altered the ability of GluA1 to recognize other AMPAR subunits. By contrast, the presence of GluA1N63Q did not affect the cell surface expression levels of GluA2 or its ability to form homotetramers [20], further supporting the notion that GluA2 is more able to recognize and bind to other AMPAR subunits, although not sufficient to promote GluA1N63Q ER-exit. Interestingly, GluA2 does not contain an N-glycosylation site homologous to GluA1N63 (Figure S1). Thus, N-glycosylation at GluA1N63 may reduce its ability to recognize other subunits and drive GluA2 ER-exit.

In this study, we focused on the regulation of ER-exit of GluA1 and GluA2 by certain N-glycans. Nevertheless, it is also important to identify the role of N-glycan in AMPAR channel function after being transported on the cell surface. We have previously shown that AMPAR channels composed of GluA1N363 and GluA2WT have normal channel activity [20]. However, since GluA1N63 is retained intracellularly, it is challenging to investigate the role of N-glycan at N63 in AMPAR channel function. Additionally, it remains unclear which N-glycans are required for proper AMPAR channel function. The role of N-glycans of GluN1, an NMDA-type glutamate receptor subunit, has been extensively investigated using nuclear magnetic resonance and molecular dynamics analyses. N-glycans on the ligand-binding region of GluN1 have been shown to directly interact with nearby peptides and N-glycans, maintaining its structure and regulating its glycin-binding ability [28,29]. Moreover, since AMPARs are tetrameric ion channels, stoichiometry studies are needed to determine how many and which N-glycans of the four subunits are necessary to maintain AMPAR function.

In conclusion, we found that N-glycans at GluA1N63, GluA1N363, and GluA2N370 have distinct roles in the ER-exit of homo-oligomers. Similar to what has been previously shown for hetero-oligomers, AMPAR subunit trafficking is strictly regulated by specific N-glycans.

## 4. Materials and Methods

### 4.1. Plasmids

All expression fragments were cloned into the pCDNA3.1B vector (Thermo Fisher Scientific, Carlsbad, CA, USA). GluA1-GFP/pCDNA3.1B and GluA2-GFP/pCDNA3.1B were constructed as previously described [30]. For the construction of HA-GluA1-myc/pCDNA3.1B and HA-GluA2-myc/pCDNA3.1B plasmids, NheI recognition sites were introduced to 12 base pairs downstream of the signal peptide in the plasmids GluA1/pcDNA3.1B [17] or GluA2/pcDNA3.1B [16] using PCR mutagenesis. Subsequently, the HA sequence was created by primer annealing (5’-ctagctacccatacgatgttccagattacgctg-3’ and 5’-ctagcagcgtaatctggaacatcgtatgggtag-3’) and cloned into the plasmids HA-GluA1/pcDNA3.1B and HA-GluA2/pcDNA3.1B. Finally, HA-GluA1 and HA-GluA2 cDNAs were amplified by PCR using the following primer pairs: 5′-tttaagcttatggccttaccagtgaccgc-3′ and 5′-gtttctagaaatcctgtggctcccaag-3′ (for HA-GluA1) and 5′-tttgcggccgcatgcaaaagattatgcatatttc-3′ and 5′-aggtctagaattttaacactctcgatgcc-3′ (for HA-GluA2). The PCR products were cloned into the HindIII-XbaI and NotI-XbaI sites of pcDNA3.1B. Different GluA1 and GluA2 mutants, in which the asparagine (Asn) residue in the consensus sequence (N-X-S/T) was mutated to glutamine (Gln), were constructed using the QuikChange Lightning Site-Directed Mutagenesis Kit (Agilent Technologies, Santa Clara, CA, USA) and the following primers: GluA1N63Q, 5′-gcttccccagatcgatattgtgcagatcagcgacagctttgag-3′; GluA1N257Q, 5′-gacaggtttccaactggtgcagtacacagacacgatcccagcc-3′; GluA1N363Q, 5′-cgagaaagggcgccggacccagtacaccctccatgtgatcg-3′; GluA2N256Q, 5′-gctgaaaattcagtttggaggagcacaggtctctggatttcag-3′; and GluA2N370Q, 5′-ccagaacggaaaacgaatacagtacacaattaacatcatggagc-3’; the respective reverse complementary primers were also used.

### 4.2. Cell Culture and Transfection

HEK293 cells purchased from the American Type Culture Collection (Manassas, VA, USA) and cultured in Dulbecco’s modified eagle medium (Nacalai Tesque, Kyoto, Japan) supplemented with 10% (v/v) fetal bovine serum (Sigma-Aldrich, St. Louis, MO, USA). HEK293 cells were authenticated by PowerPlex16 STR (Promega, Madison, WI, USA). Cells were transfected with cDNAs in Opti-MEM (Gibco, Gaithersburg, MD, USA) using Lipofectamine 2000 (Thermo Fisher Scientific), according to the manufacturer’s instructions. For co-expression experiments, cells were transfected with equal amounts of the two cDNAs (Figure 3 and Figure 4; Figure S2), aiming to express similar levels of the two subunits.

### 4.3. Immunostaining

Immunostaining of the cell surface and intracellular proteins was performed as previously described [27]. To detect the cell surface subunits (Figure 1 and Figure 2), we incubated cells at 37 °C for 2 h with an anti-GluA1 monoclonal antibody (mAb) (a kind gift from Prof. Richard L. Huganir, Johns Hopkins University) or anti-GluA2 mAb (Cat# 32-0300, Thermo Fisher Scientific). After washing with phosphate-buffered saline (PBS), cells were fixed with 4% paraformaldehyde (PFA) in PBS at room temperature for 15 min. Subsequently, cells were stained with an Alexa Fluor 546-conjugated anti-mouse IgG secondary antibody (Cat# A10036, Thermo Fisher Scientific).

To detect the cell surface HA-tagged subunits (Figure 3 and Figure 4; Figure S2), we incubated cells at 37°C for 2 h with a DyLight550-conjugated anti-HA mAb (Cat# ab117502, Abcam, Cambridge, UK). After washing with PBS, cells were fixed with 4% PFA in PBS at room temperature for 15 min, followed by permeabilization with 0.1% Triton X-100 in PBS. Subsequently, cells were incubated with anti-myc polyclonal antibody (pAb) (Cat# ab9106, Abcam) or anti-GluA2 pAb (Cat# ADI-905-414-1, Enzo Life Sciences, Farmingdale, NY, USA) in PBS containing 3% bovine serum albumin (BSA; Nacalai Tesque) at room temperature for 1 h. After washing with PBS, cells were stained with an Alexa Fluor 405-conjugated anti-rabbit IgG secondary antibody (Cat# ab175651, Abcam). Images were acquired using a FluoView imaging system (Olympus, Tokyo, Japan), and cell surface expression levels were quantified using ImageJ software. Statistical significance was determined using the Tukey’s multiple comparison test; data were analyzed using OriginPro (OriginLab, Northampton, MA, USA).

## Supplementary Materials

The following are available online at https://www.mdpi.com/1422-0067/21/14/5101/s1.
