# Supplementary material for: Distinct Cell Surface Expression Patterns of N-Glycosylation Site Mutants of AMPA-Type Glutamate Receptor under the Homo-Oligomeric Expression Conditions
Source: Int J Mol Sci. 2020 Jul 19;21(14):5101. doi: 10.3390/ijms21145101 (PMC7404163; doi:10.3390/ijms21145101)
Supplement: Supplementary file 1 [file ijms-21-05101-s001.pdf]

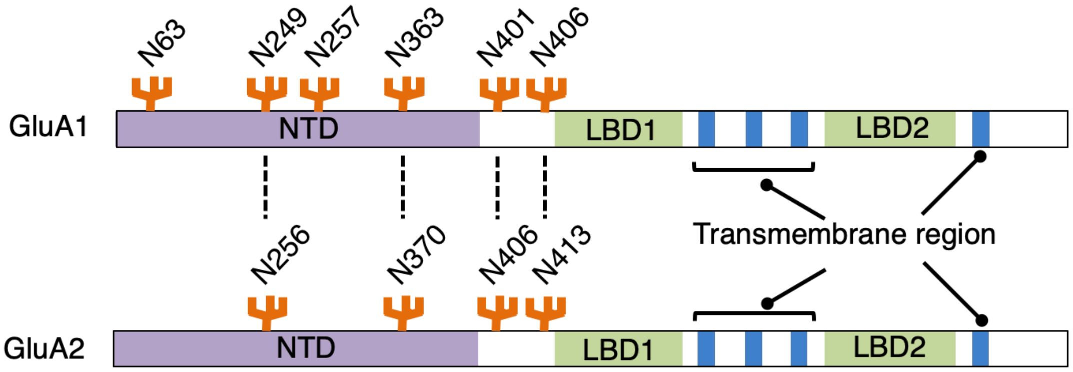

Supplementary Figure 1. N-glycosylation sites in GluA1 and GluA2.

Schematic representation of GluA1 and GluA2. GluA1 and GluA2 contain six and four N-glycosylation sites in their extracellular domains, respectively. The GluA2 N-glycosylation sites N256, N370, N406, and N413 are homologous to the GluA1 N-glycosylation sites N249, N363, N401, and N406 on GluA1, respectively. The ligand-binding domains of GluA1 and GluA2 are comprised of two extracellular domains, indicated as LBD1 and LBD2, respectively. The transmembrane regions are shown in light blue. NTD, N-terminal domain.

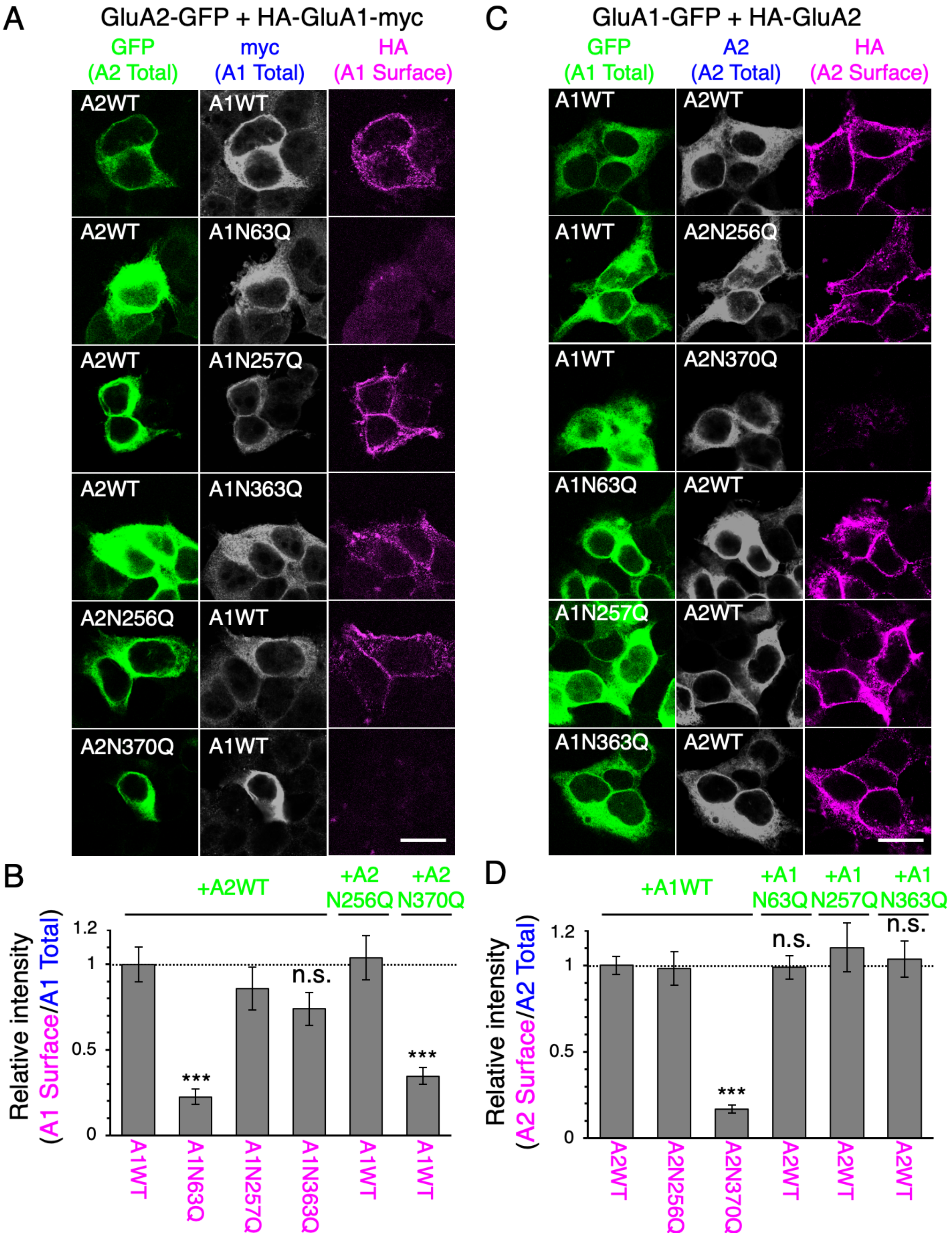

Supplementary Figure 2. Distinct expression patterns of N-glycosylation mutants.

(A) Representative immunostaining images showing the cell surface GluA1 WT and mutants (N63Q, N257Q, and N363Q) tagged with HA and myc (at the N- and C-terminus, respectively) in HEK293 cells co-expressing GFP-tagged GluA2 WT or mutants (N256Q and N370Q). Images

were acquired at 24 h post-transfection. Surface GluA1 was labeled using an anti-HA mAb, and total GluA1 was detected using an anti-myc pAb. (B) Relative cell surface levels of HA-GluA1-myc WT and mutants in cells co-expressing GluA2-GFP WT and mutants. The fluorescence intensity of HA was normalized to that of myc and heterooligomeric combination of WTs at 24 h post-transfection. (C) Representative immunostaining images showing the cell surface GluA2 WT and mutants (N256Q and N370Q) tagged with HA in HEK293 cells co-expressing GFP-tagged GluA1 WT or mutants (N63Q, N257Q, and N363Q). Images were acquired at 24 h post-transfection. Surface GluA2 was labeled using an anti-HA mAb and total GluA2 was detected using an anti-GluA2 pAb. Note that HA-GluA2 was used in this analysis because HA-GluA2 exhibited more effective expression than HA-GluA2-myc. However, HA-GluA2-myc was used in **Figure 4** to distinguish between different GluA2 subunits. (D) Relative cell surface levels of HA-GluA2 WT and mutants in cells co-expressing GluA1-GFP WT and mutants. The fluorescence intensity of HA was normalized to that of total GluA2 (labeled with anti-GluA2 pAb) and heterooligomeric combination of WTs at 24 h post-transfection. ***, $p < 0.001$; n.s., $p > 0.05$. Error bars represent the SEM. Scale bar, 20 µm.
